# Histological and Nanomechanical Properties of a New Nanometric Hydroxiapatite Implant Surface. An In Vivo Study in Diabetic Rats

**DOI:** 10.3390/ma13245693

**Published:** 2020-12-13

**Authors:** Paula G. F. P. Oliveira, Paulo G. Coelho, Edmara T. P. Bergamo, Lukasz Witek, Cristine A. Borges, Fábio B. Bezerra, Arthur B. Novaes, Sergio L. S. Souza

**Affiliations:** 1 Department of Oral and Maxillofacial Surgery and Periodontology, School of Dentistry of Ribeirao Preto, University of Sao Paulo, Av do Café, s/n., Ribeirao Preto, SP 14040-904, Brazil; paulagpessoa@yahoo.com.br (P.G.F.P.O.); cdaborges@gmail.com (C.A.B.); fabiobezerra@cenior.com.br (F.B.B.); novaesjr@forp.usp.br (A.B.N.J.); 2 Hansjorg Wyss Department of Plastic Surgery, New York University School of Medicine, New York, NY 10010, USA; pc92@nyu.edu; 3 Department of Biomaterials and Biomimetics, New York University College of Dentistry, New York, NY 10010, USA; lukasz.witek@nyu.edu; 4 Departments of Mechanical and Aerospace Engineering, New York University Tandon School of Engineering, 433 1st avenue,8th floor, Brooklyn, New York, NY 10010, USA; 5 Department of Prosthodontic and Periodontology, Bauru School of Dentistry, University of Sao Paulo, Av. Octavio Pinheiro Brisolla9-75, Bauru, SP 17012-901, Brazil; edmaratatiely@gmail.com; 6 Departments of Biomedical Engineering, New York University Tandon School of Engineering, 433 1st avenue, 8th floor, Brooklyn, New York, NY 10010, USA

**Keywords:** diabetes mellitus, dental implants, histology

## Abstract

Implant therapy is a predictable treatment to replace missing teeth. However, the osseointegration process may be negatively influenced by systemic conditions, such as diabetes mellitus (DM). Microtopography and implant surface developments are strategies associated to better bone repair. This study aimed to evaluate, in healthy and diabetic rats, histomorphometric (bone to implant contact = %BIC; and bone area fraction occupancy = %BAFO) and nanomechanical (elastic modulus = EM; and hardness = H) bone parameters, in response to a nanometric hydroxyapatite implant surface. Mini implants (machined = MAC; double acid etched = DAE, and with addition of nano-hydroxyapatite = NANO) were installed in tibias of healthy and diabetic rats. The animals were euthanized at 7 and 30 days. NANO surface presented higher %BIC and %BAFO when compared to MAC and DAE (data evaluated as a function of implant surface). NANO surface presented higher %BIC and %BAFO, with statistically significant differences (data as a function of time and implant surface). NANO surface depicted higher EM and H values, when compared to machined and DAE surfaces (data as a function of time and implant surface). Nano-hydroxyapatite coated implants presented promising biomechanical results and could be an important tool to compensate impaired bone healing reported in diabetics.

## 1. Introduction

The benefit of implants therapy is associated to osseointegration, which is described as a bone-to-implant contact without interposition of any other tissue [1]. Currently, the use of implants has been considered a satisfactory alternative to substitute teeth due to biocompatibility, esthetics, and a predictable prognosis [2]. According to Albrektson et al., 1981, some requisites are decisive for achieving osseointegration, including the biocompatibility of the material, surgical technique, implant design, condition of applied loads after implant placement, implant surface quality, and the site where the implants are installed [3]. Nonetheless, there are risk elements, which can affect and harm this process.

Diabetes mellitus (DM) is defined as a ‘‘group of metabolic diseases characterized by hyperglycemia resulting from disturbances in insulin secretion, action or both’’ [4]. Being an important health problem worldwide [5]. In this disease there is a notably high rate of periodontitis and tooth absence [6], and is considered a limiting condition to treatment with implants, due to delayed wound healing [7], incidence of microvascular disease [8], and debilitated response to infection [9]. Therefore, DM remains a relative contraindication for implant therapy [10]: patients lacking good glycemic control might not be considered appropriate for implant treatment, while well-controlled diabetic patients may be [11].

The osteoblastic activity in diabetic individuals is inhibited due the persistent hyperglycemia. Additionally, this scenario modifies a parathyroid hormone response, which controls metabolism of Ca and P [12], declines collagen formation during callus formation [13], induces apoptosis in lining cells of bone [14], and increases osteoclastic activity [15], due to persistent inflammatory response. Additionally, produces adverse effect on bone matrix and reduces growth and accumulation of extracellular matrix [16]. This results in a decrease of bone repair, which is detected in animal studies [17].

Therefore, DM remains a relative contraindication for implant therapy [10]: well-controlled diabetic patients may be considered appropriate for implant therapy, while diabetic patients lacking good glycemic control may not [11].

The osteoblastic activity in diabetic individuals is inhibited due the persistent hyperglycemia. Additionally, this scenario modifies the response of parathyroid hormone, which regulates metabolism of Ca and P [12], decreases collagen formation during callus formation [13], induces apoptosis in lining cells of bone [14], and increases osteoclastic activity [15], due to a persistent inflammatory response. It also produces an adverse effect on the bone matrix and reduces growth and accumulation of the extracellular matrix [16]. This results in a decrease of bone repair, which is detected in animal studies [17].

Current developments in the surface of osseointegrated implants has been suggested to improve osseointegration in individuals with metabolic disorders that can impair bone healing [18]. Surface topography and chemical composition are involved in the early stages of wound healing, and can alter cell behavior and tissue attachment to the implanted material [19]. The surface modification can increase the response of the bone tissue around the implant, both quantitatively (stimulate the healing process) and qualitatively (improving the quality of newly formed bone [20]). Osseointegration is directly associated to the degree of surface roughness, and can also be influenced by physical and chemical surface properties [21]. An important parameter is the bone-to implant contact (BIC), which influences the success and survival rates in implant therapy [22].

Jimbo et al., 2012 evaluated 20 titanium implants inserted into rabbit femurs, 10 with a hydroxyapatite (HA) nanoparticle surface and 10 grit-blasted, acid-etched, and heat-treated [23]. The animals were sacrificed 3 weeks after implant insertion. The nanomechanical properties of the surrounding bone were evaluated by nanoindentation. While both implants show similar BIC, the nanoindentation revealed that the tissue quality was expressively improved around the HA coated implants. It suggests that nanomechanical evaluation enable one to analyze the bone mechanical characteristics in the condensed scale level, without external influences [24].

Animal reports have revealed that DM could negatively affect implant osseointegration process [25,26]. Ajami et al., 2014, revealed that early stages of peri-implant healing (mainly osteoconduction and new bone formation) was impacted by chronic hyperglycemia, which consequently affects long-term endosseous implant stability, and that hyperglycemia-caused deficient implant integration could be minimized by using nanotopographically complex implants [27]. Furthermore, qualitative differences in newly formed bone were also reported: bone around implants was described as immature and less organized in uncontrolled diabetic rats than in non-diabetic controls [25,26].

Thus, the purpose of this study was to analyze, in healthy and diabetic rats, the response of a new topography implant surface (nano-hydroxyapatite coating), concerning histomorphometric bone regeneration (bone to implant contact—%BIC and bone area fraction occupancy—%BAFO), and nanomechanical properties (elastic modulus—EM, and hardness—H).

## 2. Materials and Methods

### 2.1. Animals

Seventy-two adult male Wistar rats (250–300 g) were included in the study. The animals were treated according to the guidelines for animal care, were kept in plastic cages with free access to water and a standard diet. For 7 days, before the surgeries, the rats were acclimated to the laboratory environment. The rats were randomly selected to one of the experimental groups (diabetes mellitus or control). The protocol was approved by The Ethics Committee on Animal Experimentation at the school of Dentistry of Ribeirao Preto, University of Sao Paulo—FORP/USP (protocol 2014.1.1083.58.4).

### 2.2. Induction of Diabetes Mellitus

Diabetes was induced by a single intraperitoneal (i.p.) injection of streptozotocin (STZ, 60 mg/kg, Sigma, St. Louis, MO, USA), which was dissolved in 0.2 mL citrate buffered solution (0.01 M, pH 4.5; *n* = 36). Healthy group received a vehicle i.p. injection at a volume of 1 mL/kg (*n* = 36). A puncture in the distal tail of the animals was performed at the days of implantation and euthanasia, to collect blood for glucose levels monitoring. Blood glucose was instantly measured with a glucometer (Accu-Check Active monitoring system, Roche Diagnostics, Rotkreuz, Zug, Switzerland). Only animals with glucose levels higher than 300 mg/dL were considered diabetic and included in the research [28].

### 2.3. Surgical Procedures

General anesthesia was induced by administering an injection of 100–130 μL/100 g of animal weight in a 4:3 combination of ketamine (74.1 mg/g of 10% ketamine hydrochloride; Agener, União Química Farmacêutica Nacional S/A, Embu-Guaçu, Sao Paulo, Brazil) and xylazine (11.2 mg/kg dopaser; Calier Laboratories S/A, Catalonia, Barcelona, Spain). Then, trichotomy and antisepsis were performed with iodine solution 10% (Rioquimica Ind. Farmaceutica, Sao Paulo, Brazil) at the medial side of the left tibia.

**Figure 1:**
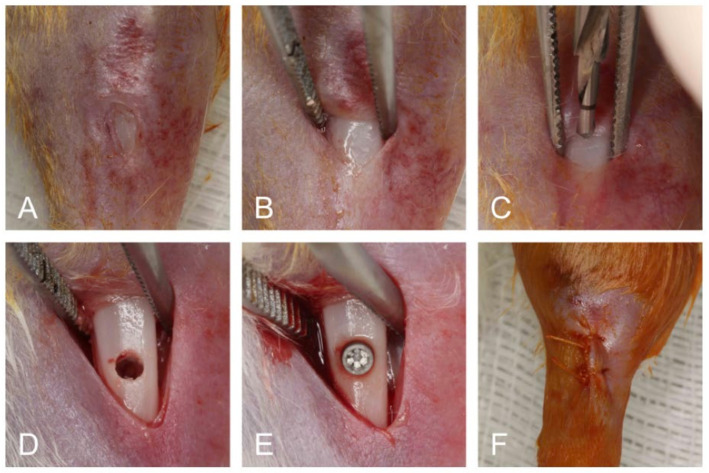
Surgical procedures (**A**–**F**). Initial aspect of tibia after trichotomy and antisepsis (**A**). Exposed bone tissue after full-thickness flap (**B**). Osteotomy being performed (**C**). Bone before implant placement (**D**). Mini implant installed (**E**). Flap sutured (**F**).

A 15-mm incision was done using a type 15C scalpel blade (Swann-Morton, Sheffield, UK) on the medial side of the left tibia. The soft tissues were dissected to exposure bone, which was then copiously washed with sterile saline solution (0.9%) and drilled with a 2.0 mm pilot drill (SIN implantes, Sao Paulo, Sao Paulo, Brazil) using an electric implant motor (Dentscler, Ribeirao Preto, Sao Paulo, Brazil) at 1000 rpm. There was constant sterile saline solution (0.9%) irrigation during the perforation. A specially designed titanium implant (2.7 mm in length and 1.4 mm in diameter—SIN implantes, Sao Paulo, Sao Paulo, Brazil) was randomly installed in the left tibia using a hand driver key (SIN implantes, Sao Paulo, Sao Paulo, Brazil). The flaps were sutured using 5-0 coated Vicryl- suture (Vicryl Ethicon 5.0, Johnson Prod., São José dos Campos, Sao Paulo, Brazil; Figure 1).

Subsequently, the animals were monitored every day to evaluated post postoperative complications. All animals received a single dose of anti-inflammatory (ketoprofen 0.05 mg/kg), and antibiotic (penicillin 24.000 IU/kg).

### 2.4. Experimental Groups

The seventy-two animals were randomly divided into six experimental groups of twelve rats each, and the groups were defined as follows: G1—machined mini implants installed in healthy rats; G2—double acid etched (DAE) mini implants installed in healthy rats; G3—nano-hydroxyapatite (NANO HA) covered mini implants installed in healthy rats; G4—machined mini implants installed in diabetic rats; G5—DAE mini implants installed in diabetic rats; and G6—NANO HA covered mini implants installed in diabetic rats.

Seven and 30 days after implants placement the rats were euthanized (36 animals in each period, 18 healthy and 18 diabetics, 6 from each group) by the intraperitoneal administration of a lethal dose (150 mg/kg) of sodium thiopental (Thiopentax, Cristalia, Sao Paulo, Brazil). The left tibia was removed and fixed in 10% neutral formalin for 48 h and the specimens were histologically processed. Histological parameters bone to implant contact (%BIC) and bone area fraction occupancy (%BAFO); and nanomechanical properties: elastic modulus (EM) and hardness (H) were evaluated.

### 2.5. Implant Design

**Figure 2:**
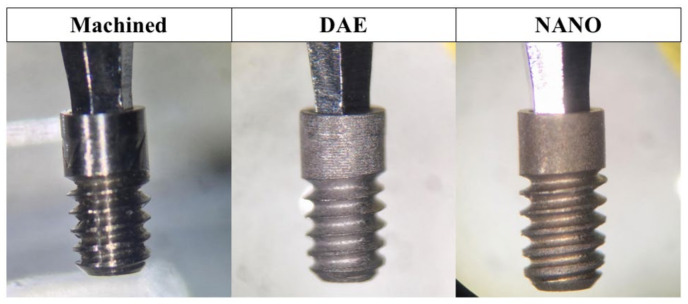
Macrogeometry of machined, DAE, and NANO implants used in the study.

This study investigated commercially pure titanium implants (grade 4). All the implants had the same design and geometry and the surfaces evaluated were machined, double acid etching (DAE), and nano-hydroxyapatite coating (NANO; Figure 2).

#### 2.5.1. Machined Surface

The machined implants did not receive any surface treatment. They were manufactured from commercially pure titanium (grade 4) cylindrical bars. During the machining process, the implants were inspected for their critical dimensional characteristics, shape/position, surface finish, and mechanical requirements. After that, they received automated prewashing by centrifugal disc units, and hygiene process, carried out inside controlled rooms (clean room), in high performance automated cleaning systems (ultrasonic cleaning systems, Elma Schmidbauer GmbH, Singen, Germany). Then, they were packed by an automated process and sterilized for use in the study.

#### 2.5.2. DAE Surface

A machined implant surface received baths of nitric acid followed by sulfuric acid, leading to the microcorrosion of the surface. The resulting surface was called DAE.

#### 2.5.3. NANO Surface

For the NANO surface treatment, a DAE implant surface was processed according to Kjellin and Andersson 2012 [29]. Succinctly, coating liquid containing nanohydroxyapatite crystals was applied on top of the implant to be coated, and the implant was placed on a spin coater device. Then, for homogenization of the liquid over the entire surface, the implant was rotated at 2600 rpm for 3 s; after that, it was allowed to dry at 10 min in room temperature. Finally, the implant was placed in an oven at 450 °C for 5 min, for sintering and stable adhesion of the HA crystals.

DAE and NANO mini implants were packed and sterilized as previously described for the machined surface.

### 2.6. Histologic Sectioning and Histomorphometry

Briefly, the tibia-implant blocks were fixed in 10% formalin buffered with 0.1 M sodium cacodylate, pH 7.3, for 48 h and transferred to a solution of 70% ethanol for 72 h. After dehydration, tibias were embedded in Hard Grade LR White resin (London Resin Company, London, UK) and sectioned using the Exakt Cutting System (Exakt, Norderstedt, Hamburg, Germany). Then, the Exakt Grinding System (Exakt, Norderstedt, Hamburg, Germany) was used to polish the longitudinal sections, which were mounted on acrylic slides. The final thickness of the sections was 20 μm, and then they stained with Stevenel’s blue and Alizarin red and scanned via an automated slide scanning system and computer software (Aperio Technologies, Vista, CA, USA).

For histomorphometric evaluation, an imaging analysis software (ImageJ, NIH, Bethesda, MD, USA) was used to quantify and evaluate osseointegration parameters around peri-implant surface. The dependent variables of the present study were percentage of bone-to-implant contact (%BIC) and bone area fraction occupancy (%BAFO). %BIC was selected because it measures the percentage of bone along with implant surface perimeter, quantifying the degree of osseointegration derived from initial bone stabilization. %BAFO measures the percentage of bone (newly formed and non-vital autografted/native bone due to instrumentation) within the implant threads, evaluating the degree of osseointegration derived from secondary stability by [30,31,32]. All evaluations were performed in a blinded manner.

### 2.7. Nanoindentation Assay

The nanoindentation test was performed as previously described by Coelho et al., 2014 [24]. Briefly, the resin blocks were processed in the same manner as the histological sections, and then received further polishing with diamond suspensions of 9–1 μm particle size (Buehler, Lake Bluff, IL, USA) for scratch removal. Nanoindentation (*n* = 30 per specimen) were performed with a nanoindenter (Hysitron TI 950, Minneapolis, MN, USA) equipped with a Berkovich diamond three-sided pyramid probe [33]. A loading profile was developed with a peak load of 300 μN at a rate of 60 μN/s, followed by a holding time of 10 s and an unloading time of 2 s. The extended holding period is necessary for bone to relax to a more linear response, so that no tissue creep effect occurred in the unloading portion of the profile. Therefore, from each indentation, a load–displacement curve was obtained [34].

For each specimen, mechanical testing was carried out within the threaded regions (cortical area) between the first and second plat eau or the initial set of interplateau spaces containing novel bone formation. The specimen was discarded if does not present cortical bone within any of the interplateau regions. A light microscope (Hysitron, Minneapolis, MN, USA) was used to initially detect via imaging the bone tissue within these regions [35]. Indentations (*n* = 30/implant) were performed in the identified regions of interest within each plateau region.

From each generated load–displacement curve, the reduced modulus Er (GPa) and hardness H (GPa) of cortical bone tissue were computed via the Hysitron TriboScan software using the following formulae, respectively:

(1) $$E_{r\;}=\frac{\sqrt{\pi }}{2\sqrt{A\left(h_{c}\right)}}\times \;S;\;H=\;\frac{\mathrm{Pmax}}{A\left(h_{c}\right)}$$

where S is the stiffness, hc is the contact depth, Pmax is the maximum applied force (300 μN), and A(hc) is the contact area computed from the TriboScan software (version 9.2.12.0) utilizing the area function with respect to the contact depth. Through the reduced modulus Er, the corresponding elastic modulus Eb (GPa) may be calculated using the following equation:

(2) $$\frac{1}{E_{r}}=\frac{1-{\;v}_{b}^{2}}{E_{b}}+\frac{1-{\;v}_{i}^{2}}{E_{i}}$$

where vb (0.3) is the Poisson’s ratio for cortical bone, Ei (1140 GPa) is the elastic modulus of the indenter, and vi (0.07) is the Poisson’s ratio for the indenter [36,37]. This methodology [38] has been shown successful in determining the mechanical properties of bone through nanoindentation [23,33,39,40].

### 2.8. Statistical Analysis

The histomorphometric data (percentage of bone-to-implant contact = %BIC and bone area fraction occupancy = %BAFO) and nanomechanical properties (elastic modulus = EM and hardness = H) are presented as a function of mean values with the corresponding 95% confidence interval (mean ± 95% CI). Preliminary analyses of %BIC, %BAFO, EM, and H have shown indistinguishable variances (Levene’s test, all *p* > 0.25). %BIC, %BAFO, EM, and H data were collected and aligned along a linear mixed model with fixed factors of time (7 and 30 days), systemic condition (healthy and diabetic), and surface chemistry/texture modifications (machined, DAE, and NANO) at a random intercept. After administering a significant omnibus test, post-hoc comparison of the experimental groups means was gathered using a Tukey’s test. The analysis was accomplished using SPSS (IBM SPSS 23, IBM Corp., Armonk, NY, USA).

## 3. Results

The surgical procedures and follow-up revealed no complications regarding technical conditions or post-operative infections. Post-operative healing was uneventful. No adverse events were identified, and a clinically healthy soft tissue aspect was observed through the study.

### 3.1. Histomorphometric Analysis

**Figure 3:**
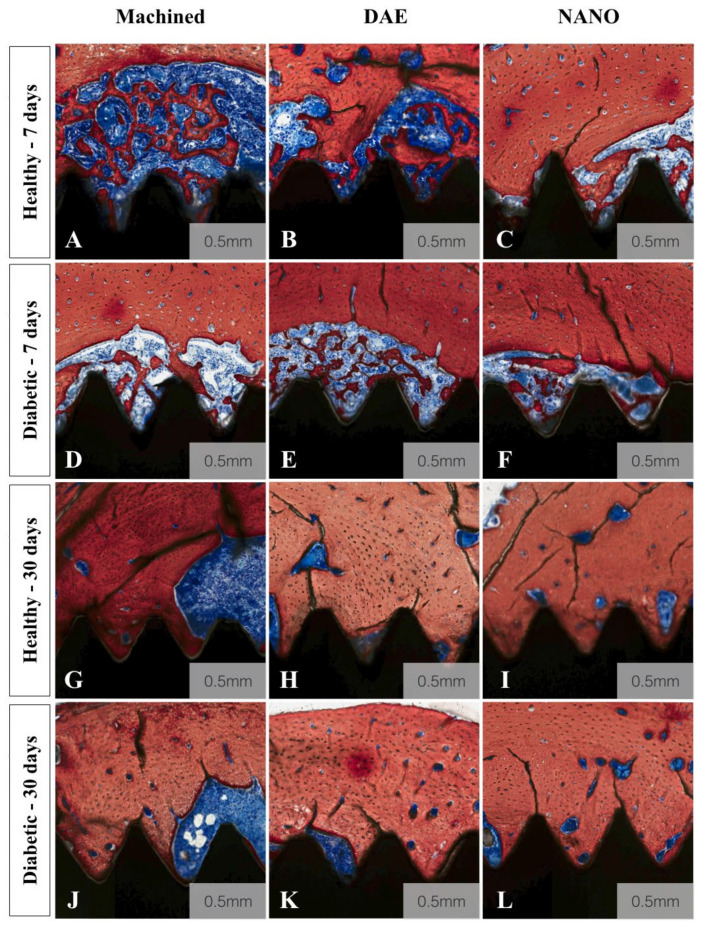
Histologic micrographs of all experimental groups (**A**–**L**). Stevenel’s blue Alizarin red stained histologic micrographs of healthy (**A**–**C**,**G**–**I**) and diabetic (**D**–**F**,**J**–**L**) groups for machined (**A**,**D**,**G**, and **E**), DAE (**B**,**E**,**H**, and **K**), and NANO coated (**C**,**F**,**I**, and **L**) implant surface treatments. All images confirmed successful osseointegration, irrespective of time, implant type, and systemic condition. Overall, a notable difference was observed between 7 and 30 days. With regards to implant type, the NANO group demonstrated more intimate bone to implant interlocking when compared to machined and DAE. Additionally, at 30 days, NANO coating implants micrographs depicted increased remodeling sites and new bone formation compared to 7 day images.

A qualitative evaluation of the histologic micrographs of all groups is depicted in Figure 3A–L. All images confirmed successful osseointegration, irrespective of time, implant type, and systemic condition. Overall, a notable difference was observed between 7 and 30 days. With regards to implant type, the NANO group demonstrated more intimate bone to implant interlocking when compared to machined and DAE. Additionally, at 30 days, NANO coating implants micrographs depicted increased remodeling sites and new bone formation compared to 7 day images.

#### 3.1.1. Bone to Implant Contact (%BIC)

**Figure 4:**
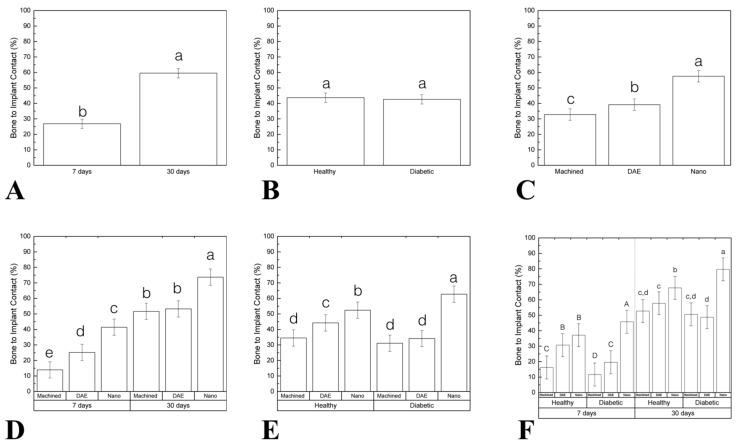
(**A**–**F**) Graphs representing the percentage of bone to implant contact (%BIC) differently arranged as a function of study factors (mean and 95%CI). Significant difference was seen between 7 and 30 days in vivo (**A**). No statistically significant differences were found when comparing the healthy and diabetic group (data as function of systemic condition) (**B**). When implant surface was considered, NANO group exhibited higher %BIC relative to machined and DAE groups (data as a function of implant surface) (**C**). Data evaluation as a function of time and implant surface highlighted the superiority of the NANO group at 30 days compared to others (**D**). When %BIC was evaluated as a function of the systemic condition and surface, diabetic NANO group depicted a significant statistical difference (**E**). Evaluating all factors: time, systemic condition, and surface treatment the results revealed that NANO surface presented the best results in the diabetic group (7 and 30 days) (**F**). Identical letters indicate no significant difference among groups.

Correlating to histologic analysis, histomorphometric measurements of the percentage of bone to implant contact (%BIC) as a function of time revealed a statistical significant difference between 7 (25.32 ± 2.61) and 30 (59.69 ± 2.61) days (*p* < 0.001; Figure 4A). No statistically significant differences were found when healthy (43.03 ± 2.61) and diabetic (41.96 ± 2.61) groups were compared (data as function of systemic condition; *p* = 0.632; Figure 4B). When implant surfaces were considered, NANO group (57.39 ± 3.20) exhibited higher %BIC relative to machined (32.35 ± 3.20) and DAE (37.74 ± 3.20) groups (data as a function of implant surface; *p* < 0.001; Figure 4C). Data evaluation as a function of time and implant surface highlighted the superiority of NANO group at 30 days (74.13 ± 4.53) compared to others (machined 7 days = 13.62 ± 4.53, DAE 7 days = 21.62 ± 4.53, NANO 7 days = 40.65; machined 30 days = 51.07 ± 4.53 DAE 30 days = 53.87 ± 4.53; *p* < 0.001; Figure 4D). When %BIC was evaluated as a function of systemic condition and surface, diabetic (62.58 ± 4.53) NANO group depicted a significant statistical difference (*p* < 0.001; Figure 4E). Evaluating all factors: time, systemic condition, and surface treatments, the results revealed that NANO surface presented the best results in diabetic 7 (35.94 ± 6.40) and 30 days (79.80 ± 6.40; *p* < 0.001; Figure 4F).

#### 3.1.2. Bone Area Fraction Occupancy (%BAFO)

**Figure 5:**
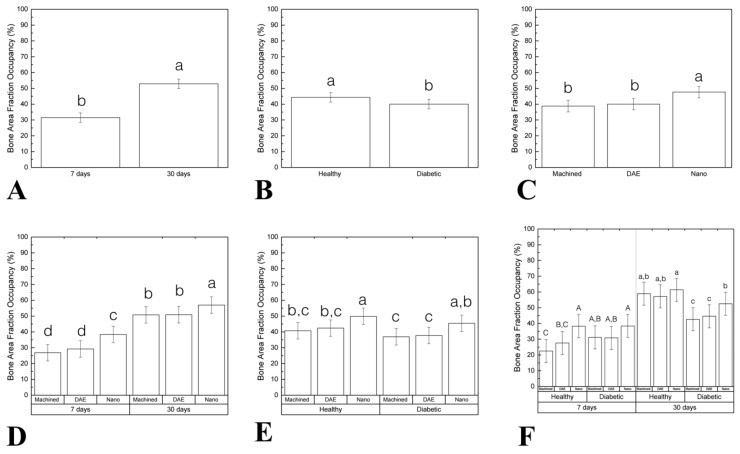
(**A**–**F**) Graphs representing the percentage of bone area fraction occupancy (%BAFO) differently arranged as a function of study factors (mean and 95% CI). Significant difference was seen between 7 and 30 days in vivo (**A**). Statistically significant difference was found when comparing the healthy and diabetic group (data as function of systemic condition) (**B**). When implant surface was considered, the NANO group exhibited higher %BAFO relative to machined and DAE groups (data as a function of implant surface) (**C**). Data evaluation as a function of time and implant surface highlighted the superiority of NANO group at 30 days compared to others (**D**). No statistically significant differences were found when %BAFO was evaluated as a function of systemic condition and surface (**E**). Evaluating all factors (time, systemic condition, and surface treatment) the results revealed that NANO surface presented the best results at 7 days (healthy group) (**F**). Identical letters indicate no significant difference among groups.

Percentage of bone area fraction occupancy (%BAFO) as a function of the time demonstrated statistic significant difference between 7 (31.46 ± 2.99) and 30 (52.87 ± 2.99) days (*p* < 0.001; Figure 5A). Healthy group (44.31 ± 2.99) presented higher %BAFO when compared to diabetic (40.02 ± 2.99; data as a function of systemic condition; *p* = 0.048; Figure 5B). When implant surfaces were considered, NANO group (47.64 ± 3.66) exhibited higher %BAFO relative to machined (38.80 ± 3.66) and DAE (40.05 ± 3.66) groups (data as a function of implant surface; *p* = 0.002; Figure 5C). Data evaluation as a function of time and implant surfaces highlighted the superiority of the NANO group at 30 days (56.95 ± 5,18) compared to others (machined 7 days = 26.83 ± 5.18, DAE 7 days = 29.22 ± 5.18, NANO 7 days 38.34 ± 5.18, machined 30 days = 50.77 ± 5.18, DAE 30 days = 50.88 ± 5.18; *p* < 0.001; Figure 5D). No statistically significant differences were found when %BAFO was evaluated as a function of systemic condition and surface (*p* = 0.616; Figure 5E). Evaluating all factors (time, systemic condition, and surface treatment) the results revealed that NANO surface presented the best results at 7 days (healthy = 38.28 ± 7.33; *p* < 0.009; Figure 5F).

### 3.2. Nanomechanical Analysis

#### 3.2.1. Elastic Modulus (EM)

**Figure 6:**
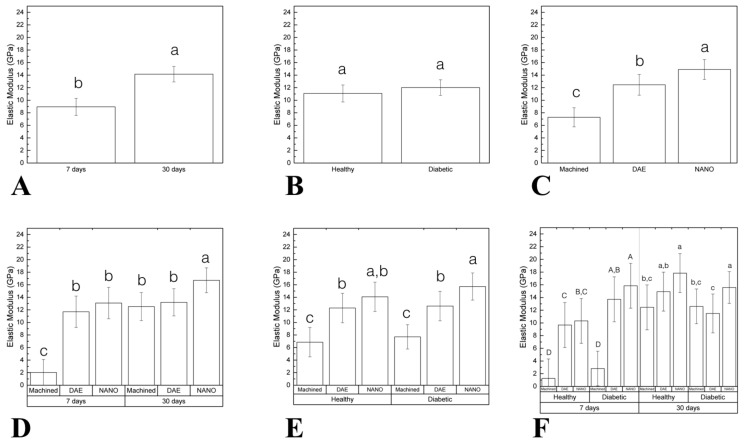
(**A**–**F**) Graphs representing elastic modulus (EM) differently arranged as a function of study factors (mean and 95% CI). A significant difference was seen between 7 and 30 days in vivo (**A**). No statistically significant difference was found when comparing the healthy and diabetic group (data as a function of systemic condition) (**B**). When the implant surface was considered, NANO group exhibited higher EM relative to machined and DAE groups (data as a function of implant surface) (**C**). Data evaluation as a function of time and implant surface highlighted the superiority of NANO group at 30 days compared to others (**D**). When EM was evaluated as a function of systemic condition and surface, diabetic NANO group depicted higher values (**E**). Evaluating all factors: time, systemic condition and surface treatment the results revealed that surface treated implants presented superior EM than machined (**F**). Identical letters indicate no significant difference among groups.

The elastic modulus (EM) as a function of time demonstrated statistic significant difference between 7 (8.94 ± 1.36) and 30 (14.14 ± 1.22) days (*p* < 0.001; Figure 6A). No statistically significant differences were found when compared healthy (11.07 ± 1.35) and diabetic (12.01 ± 1.24) groups (data as function of systemic condition; *p* = 0.308; Figure 6B). When implant surfaces were considered, NANO group (14.89 ± 1.59) exhibited higher EM relative to machined (7.28 ± 1.51) and DAE (12.45 ± 1.65) groups (data as a function of implant surface; *p* < 0.037; Figure 6C). Data evaluation as a function of time and implant surface highlighted the superiority of NANO group at 30 days (16.70 ± 1.97) compared to others (machined 30 days = 12.52 ± 2.23, DAE 30 days = 13.20 ± 2.16; *p* < 0.027; Figure 6D). When EM was evaluated as a function of systemic condition and surface, the diabetic NANO group (15.71 ± 2.16) depicted higher values (*p* < 0.001; Figure 6E). Evaluating all factors (time, systemic condition, and surface treatment) the results revealed that surface treated implants presented superior EM than machined (*p* < 0.031; Figure 6F).

#### 3.2.2. Hardness (H)

**Figure 7:**
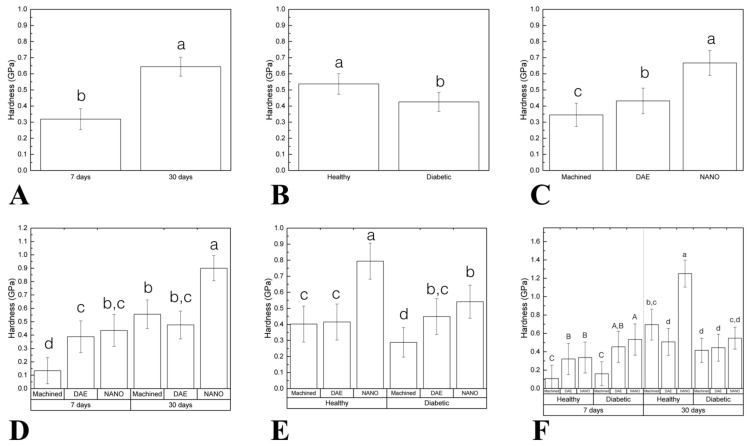
(**A**–**F**) Graphs representing hardness (**H**) differently arranged as a function of study factors (mean and 95% CI). Significant difference was seen between 7 and 30 days in vivo (**A**). Healthy group presented higher H when compared to diabetic (data as function of systemic condition) (**B**). When implant surface was considered, NANO group exhibited higher H relative to machined and DAE groups (data as a function of implant surface) (**C**). Data evaluation as a function of time and implant surface highlighted the superiority of NANO group at 30 days compared to others (**D**). When H was evaluated as a function of the systemic condition and surface, the healthy NANO group depicted higher values (**E**). Evaluating all factors: time, systemic condition, and surface treatment the results revealed that surface treated implants presented superior H than machined and that NANO healthy 30 days presented the higher values of H (**F**). Identical letters indicate no significant difference among groups.

Hardness (H) as a function of time demonstrated statistic significant difference between 7 (0.31 ± 0.06) and 30 (0.64 ± 0.05) days (*p* < 0.001; Figure 7A). Healthy group (0.53 ± 0.06) presented higher H when compared to diabetic (0.42 ± 0.05; data as a function of systemic condition; *p* = 0.015; Figure 7B). When the implant surface was considered, NANO group (0.66 ± 0.07) exhibited higher H relative to machined (0.34 ± 0.07) and DAE (0.43 ± 0.07) groups (data as a function of implant surface; *p* < 0.037; Figure 7C). Data evaluation as a function of time and implant surface highlighted the superiority of NANO group at 30 days (0.9 ± 0.09) compared to others (machined 30 days = 0.55 ± 0.10, DAE 30 days = 0.47 ± 0.10; *p* < 0.027; Figure 7D). When H was evaluated as a function of systemic condition and surface, the healthy NANO group (0.79 ± 0.11) depicted higher values (*p* < 0.002; Figure 7E). Evaluating all factors (time, systemic condition, and surface treatment) the results revealed that surface treated implants presented superior H than machined (*p* < 0.025) and that NANO healthy 30 days (1.25 ± 0.14) presented the higher values of H (Figure 7F).

## 4. Discussion

Osseointegration has been described as the close contact between bone and biomaterials at the optical microscopic level [41] with a success rate for oral rehabilitation higher than 90% [42]. An important attention has been devoted to implant design to improve host-to-implant biocompatibility and biomechanical responses. Surface treatment has been widely investigated because it is the first part to interact with the host and these changes can alter bone-implant interactions [43].

The present study evaluated, in healthy and diabetic rats, the response of a new topography implant surface (nano-hydroxyapatite coating), concerning histomorphometric bone regeneration (%BIC and %BAFO) and nanomechanical properties (elastic modulus and hardness). The postulated null hypothesis that the histological and mechanical characteristic of the bone would be enhanced due to the effect of the nanostructured HA was accepted since histomorphometric measurements and nanomechanical properties depicted statistically significant differences for the NANO group in all evaluated parameters (data as a function of implant surface).

The basic osseointegration events of hemostasis, inflammation, proliferation, and remodeling are related to being impaired by metabolically compromised health conditions, such as diabetes mellitus (DM) [25,44,45,46]. In order to increase the rate and extend of osseointegration of implants in such challenging scenarios, implant surface chemistry/texture modifications have been suggested [47]. Nonetheless, studies comparing their potential benefits are still not clear when used in a DM model [48]. In this work, bone remodeling shown to be superior around implants with treated surfaces and to a reduced level around machined implants. The outcomes recommend that bone remodeling shows to be accelerated when related to advanced, optimized, and modified implant surfaces.

The development of microscale surface topography, as dual acid-etched (DAE) implants, enhanced bone formation along with the implant surface when compared to machined [49,50]. Additionally, the emergence of chemically/topographically complex surfaces, such as bioactive nano-hydroxyapatite coated implants (NANO), provided a more suitable arrangement that better mimics natural tissue organization and facilitates interaction with tissues biomolecules and cell–cell communication during the healing process [51,52,53]. The consequences of nanostructure on bone formation in a hyperglycemic rat model revealed an increased wettability, leading to an enhanced hard tissue differentiation [46].

Percentage of BIC is an important factor to evaluate the potential of osseointegration and to compare different implants with diverse macro- and microdesigns, materials, or surface modifications [54,55]. On the histomorphometrical analysis, the NANO surface presented higher %BIC and %BAFO when compared to machined and DAE (regarding implant surface). Furthermore, through an implant-specific healing mechanism, at 30 days, the NANO surface presented higher %BIC and %BAFO than machined and DAE, with statistically significant differences (data evaluation as a function of time and implant surface). Literature findings support the evidence through previous animal studies that demonstrated NANO complex surfaces outperforming microtopography surfaces in metabolically compromised subjects [18,27,56]. The consequence of high glucose levels combined with inflammation in a titanium surface were proposed by Ramenzoni et al., 2020, including a decrease on osteoblast processes, along with the proliferation and differentiation [45].

The noteworthy results evidenced for complex implant surface designs in challenging situations, such as low-density bone sites, motivated their use for compromised systemic conditions that affect bone metabolism and osseointegration [57]. Nanoindentation of bone around implants has the prospective to clarify the qualitative features of osseointegration [23]. In our study, data evaluated as a function of time depicted the superiority of EM and H at 30 days compared to 7 days. When the data was evaluated as a function of the implant surface, NANO group presented higher EM and H than DAE and machined. Literature findings comparing the use of NANO and DAE surfaces have confirmed the superiority of the former regarding to histological parameters [57,58].

Histological analysis has been described as the “gold standard” to evaluate bone formation. However, this method has some limits. For instance, cutting and grinding the implant in bone blocks may injury the interface and influence outcomes. Two dimensional histology may not have took the actual situation due to its limited possibility of assessment, since it may depend on where the section was taken from [59]. Therefore, the lack of a microtomographic investigation can be considered a limitation of this study. Additionally, gene expression analysis could provide insights about normal cellular processes, such as osteoblast differentiation of some important bone markers, alkaline phosphatase protein expression, and extracellular matrix mineralization [60,61]. Finally, other periods of evaluation might contribute for more reliable results.

To investigate surface modifications at the nanoscale, Oliveira et al., 2020, evaluated in healthy and diabetic rats, the response of nano-hydroxyapatite coating implants through gene expression analysis and 3D evaluation using microcomputed tomography (micro-CT), and described some findings where NANO-HA surface was less affected by these adverse effects of the high glucose environment: the levels of Runt-related transcription factor 2 (Runx2), Alkaline phosphatase (Alp), Osteopontin (Opn), and Osteocalcin (OC) were better for NANO-HA group, suggesting an enhancement of osteoblast proliferation, mainly at early stages of osseointegration [62]. Additionally, the effects of nanostructured HA coated implant surface (NANO) were observed after 7 and 30 days, in healthy and diabetic animals (as a model of impaired bone healing) [62]. In the present study, using the same methodology, the results revealed nano-hydroxyapatite coated implants encouraging biomechanical outcomes, suggesting an improvement not only in quantity but also in new bone quality, which should be a significant tool to compensate impaired bone healing related to diabetes mellitus.

The coating of titanium implants with different substances is an approach that has been investigated in the literature. The outcomes from Valbonetti et al., 2019 investigating the effects of collagen type I as a coating of titanium implants on bone healing and bone formation in a rabbit model, confirms that collagen type I is one of the most efficient approaches for accelerating early osteogenesis and improving the bioactivity of titanium implant surfaces. The collagen coating probably influences cell migration, attachment, proliferation, and differentiation on the titanium implants, and could be clinically advantageous for shortening the implant healing period [63]. These results, favoring implant coating, were similar to those found in the present study.

Additionally, literature depicted previous research that demonstrated the effects of antidiabetic drugs (for example, aminoguanidine and insulin) in order to control the negative effect of diabetes mellitus on bone around the implant in rats [28,64,65]. Yildirim et al., 2020, revealed that Metformin favors the bone healing around titanium implants and enhances the osseointegration process. Bone formation ratio was high in the test animals (metformin treated rats), when compared with the controls during the four-week osseointegration period [64]. The present study did not comprise a group with the antidiabetic drug, and this could be a suggestion for futures studies evaluating NANO-HA surface.

One of the main limitations of the present study is that the implants were inserted in rat tibia, and not in maxillary or mandibular bone. Future animal studies addressing the behavior of this nano-HA surface should be conducted in larger animals, such as mini pigs, allowing the use of commercial implants (and not mini implants specially designed for the study) installed in in alveolar bone; furthermore, more realistic clinical situations, such as implant loading and masticatory function, could be part of the experiment. Another suggestion for future studies is the inclusion of a controlled diabetic group, since the present study compared healthy versus non-controlled diabetic subjects.

This report revealed an important upregulation of the mineralization process that was revealed with bone mechanical property assessment through nanoindentation and histologic parameters. The conventional techniques, such as the removal torque testing, could be affected not only by the micro but also by the macrofeatures of the implant. Furthermore, nanomechanical characterization offers a probability to purely assess the bone mechanical features in the reduced scale level without the interruption of other factors. The nanoindentation analysis is an appropriate way to take into account the anisotropic organization of bone [37] and map the range of mechanical properties of bone around dental implants. Bone mineralization process around implant design variations (e.g., instrumentation, bulk, and surface) can be evaluated. In this work, it is well-defined that the degree of mineralization (elastic modulus and hardness) of the bone in the proximity of the implant was notably improved for surface treated implants, regardless of systemic condition and time. Subsequently, the current results and literature findings support the principle regarding to the use of bioactive nanoscale surface modifications as a compensation for the negative effect of DM on osseointegration.

## 5. Conclusions

Nano-hydroxyapatite coated implants revealed encouraging biomechanical outcomes and could be considered a significant tool to compensate impaired bone healing related in diabetics. These outcomes suggest that this surface must be confirmed in human controlled trials, evaluating challenging clinical conditions.

## References

[1] Brånemark P.-I., Breine U., Adell R., Hansson B.O., Lindström J., Ohlsson Å. (1969). Intra-osseous anchorage of dental prostheses: I. Experimental studies. Scand. J. Plast. Reconstr. Surg..

[2] Geckili O., Bilhan H., Geckili E., Cilingir A., Mumcu E., Bural C. (2014). Evaluation of possible prognostic factors for the success, survival, and failure of dental implants. Implant. Dent..

[3] Albrektsson T., Branemark P.I., Hansson H.A., Lindstrom J. (1981). Osseointegrated titanium implants. Requirements for ensuring a long-lasting, direct bone-to-implant anchorage in man. Acta Orthop. Scand..

[4] American Diabetes Association (2005). Diagnosis and classification of diabetes mellitus. Diabetes Care.

[5] King H., Aubert R.E., Herman W.H. (1998). Global burden of diabetes, 1995–2025: Prevalence, numerical estimates, and projections. Diabetes Care.

[6] Oliver R.C., Tervonen T. (1993). Periodontitis and tooth loss: Comparing diabetics with the general population. J. Am. Dent. Assoc..

[7] Rothwell B.R., Richard E.L. (1984). Diabetes mellitus: Medical and dental considerations. Spéc. Care Dent..

[8] Frantzis T.G., Reeve C.M., Brown A.L. (1971). The ultrastructure of capillary basement membranes in the attached gingiva of diabetic and nondiabetic patients with periodontal disease. J. Periodontol..

[9] McMahon M.M., Bistrian B.R. (1995). Host defenses and susceptibility to infection in patients with diabetes mellitus. Infect. Dis. Clin. North Am..

[10] Michaeli E., Weinberg I., Nahlieli O. (2009). Dental implants in the diabetic patient: Systemic and rehabilitative considerations. Quintessence Int..

[11] Oates T.W., Huynh-Ba G., Vargas A., Alexander P., Feine J. (2013). A critical review of diabetes, glycemic control, and dental implant therapy. Clin. Oral Implant. Res..

[12] Santana R.B., Xu L., Chase H.B., Amar S., Graves D.T., Trackman P.C. (2003). A role for advanced glycation end products in diminished bone healing in type 1 diabetes. Diabetes.

[13] Gooch H.L., Hale J.E., Fujioka H., Balian G., Hurwitz S.R. (2000). Alterations of cartilage and collagen expression during fracture healing in experimental diabetes. Connect. Tissue Res..

[14] He H., Liu R., Desta T., Leone C., Gerstenfeld L.C., Graves D.T. (2004). Diabetes causes decreased osteoclastogenesis, reduced bone formation, and enhanced apoptosis of osteoblastic cells in bacteria stimulated bone loss. Endocrinology.

[15] Kayal R.A., Tsatsas D., Bauer M.A., Allen B., Al-Sebaei M.O., Kakar S., Leone C.W., Morgan E.F., Gerstenfeld L.C., Einhorn T.A. (2007). Diminished bone formation during diabetic fracture healing is related to the premature resorption of cartilage associated with increased osteoclast activity. J. Bone Miner. Res..

[16] Weiss R.E., Gorn A.H., Nimni M.E. (1981). Abnormalities in the biosynthesis of cartilage and bone proteoglycans in experimental diabetes. Diabetes.

[17] Lu H., Kraut D., Gerstenfeld L.C., Graves D.T. (2003). diabetes interferes with the bone formation by affecting the expression of transcription factors that regulate osteoblast differentiation. Endocrinology.

[18] Schlegel K.A., Prechtl C., Möst T., Seidl C., Lutz R., Von Wilmowsky C. (2013). Osseointegration of SL Active implants in diabetic pigs. Clin. Oral Implant. Res..

[19] Souza A.T.P., Bezerra B.L., Oliveira F.S., Freitas G.P., Trevisan R.L.B., Oliveira P.T., Rosa A.L., Beloti M. (2018). Effect of bone morphogenetic protein 9 on osteoblast differentiation of cells grown on titanium with nanotopography. J. Cell. Biochem..

[20] Junior A.B.N., De Souza S.L.S., De Barros R.R.M., Pereira K.K.Y., Iezzi G., Piattelli A. (2010). Influence of implant surfaces on osseointegration. Braz. Dent. J..

[21] Le Guéhennec L., Soueidan A., Layrolle P., Amouriq Y. (2007). Surface treatments of titanium dental implants for rapid osseointegration. Dent. Mater..

[22] Schulz M.C., Korn P., Stadlinger B., Range U., Möller S., Becher J., Schnabelrauch M., Mai R., Scharnweber D., Eckelt U. (2013). Coating with artificial matrices from collagen and sulfated hyaluronan influences the osseointegration of dental implants. J. Mater. Sci. Mater. Med..

[23] Jimbo R., Coelho P., Bryington M., Baldassarri M., Tovar N., Currie F., Hayashi M., Janal M., Andersson M., Ono D. (2012). Nano hydroxyapatite-coated implants improve bone nanomechanical properties. J. Dent. Res..

[24] Coelho P.G., Takayama T., Yoo D., Jimbo R., Karunagaran S., Tovar N., Janal M.N., Yamano S. (2014). Nanometer-scale features on micrometer-scale surface texturing: A bone histological, gene expression, and nanomechanical study. Bone.

[25] Giglio M.J., Giannunzio G., Olmedo D.G., Guglielmotti M.B. (2000). Histomorphometric study of bone healing around laminar implants in experimental diabetes. Implant. Dent..

[26] McCracken M., Lemons J.E., Rahemtulla F., Prince C.W., Feldman D. (2000). Bone response to titanium alloy implants placed in diabetic rats. Int. J. Oral Maxillofac. Implant..

[27] Ajami E., Mahno E., Mendes V., Bell S., Moineddin R., Davies J.E. (2014). Bone healing and the effect of implant surface topography on osteoconduction in hyperglycemia. Acta Biomater..

[28] Margonar R., Sakakura C.E., Holzhausen M., Pepato M.T., Alba J.R.C., Marcantonio J.E. (2003). The influence of diabetes mellitus and insulin therapy on biomechanical retention around dental implants: A study in rabbits. Implant. Dent..

[29] Kjellin P., Andersson M. (2012). Synthetic Nano-Sized Crystalline Calcium Phosphate and Method of Production. US Patent.

[30] Coelho P.G., Marin C., Granato R., Giro G., Suzuki M., Bonfante E.A. (2011). Biomechanical and histologic evaluation of non-washed resorbable blasting media and alumina-blasted/acid-etched surfaces. Clin. Oral Implant. Res..

[31] Jinno Y., Jimbo R., Tovar N., Teixeira H.S., Witek L., Coelho P.G. (2017). In vivo evaluation of dual acid-etched and grit-blasted/acid-etched implants with identical macrogeometry in high-density bone. Implant. Dent..

[32] Gil L., Sarendranath A., Neiva R., Marão H., Tovar N., Bonfante E.A., Janal M., Castellano A., Coelho P. (2017). Bone healing around dental implants: Simplified vs conventional drilling protocols at speed of 400 rpm. Int. J. Oral Maxillofac. Implant..

[33] Baldassarri M., Bonfante E., Suzuki M., Marin C., Granato R., Tovar N., Coelho P.G. (2012). Mechanical properties of human bone surrounding plateau root form implants retrieved after 0.3–24 years of function. J. Biomed. Mater. Res. Part B Appl. Biomater..

[34] Doerner M.F., Nix W.D. (1986). A method for interpreting the data from depth-sensing indentation instruments. J. Mater. Res..

[35] Butz F., Aita H., Wang C., Ogawa T. (2006). Harder and stiffer bone Osseo integrated to roughened titanium. J. Dent. Res..

[36] Hoffler C.E., Guo X.E., Zysset P.K., Goldstein S.A. (2005). An application of nanoindentation technique to measure bone tissue lamellae properties. J. Biomech. Eng..

[37] Hoffler C., Moore K., Kozloff K., Zysset P., Brown M., Goldstein S.A. (2000). Heterogeneity of bone lamellar-level elastic moduli. Bone.

[38] Lee K.-L., Sobieraj M., Baldassarri M., Gupta N., Pinisetty D., Janal M.N., Tovar N., Coelho P.G. (2013). The effects of loading conditions and specimen environment on the nanomechanical response of canine cortical bone. Mater. Sci. Eng. C.

[39] Anchieta R.B., Baldassarri M., Guastaldi F., Tovar N., Janal M.N., Gottlow J., Dard M., Jimbo R., Coelho P. (2014). Mechanical property assessment of bone healing around a titanium-zirconium alloy dental implant. Clin. Implant. Dent. Relat. Res..

[40] Jimbo R., Anchieta R., Baldassarri M., Granato R., Marin C., Teixeira H.S., Tovar N., Vandeweghe S., Janal M.N., Coelho P.G. (2013). Histomorphometry and bone mechanical property evolution around different implant systems at early healing stages: An experimental study in dogs. Implant Dent..

[41] Albrektsson T., Wennerberg A. (2004). Oral implant surfaces: Part 2-Review focusing on clinical knowledge of different surfaces. Int. J. Prosthodont..

[42] Henry P.J., Laney W.R., Jemt T., Harris D., Krogh P.H., Polizzi G., Zarb G.A., Herrmann I. (1996). Osseointegrated implants for single-tooth replacement: A prospective 5-year multicenter study. Int. J. Oral Maxillofac. Implant..

[43] Freitas G.P., Lopes H.B., Martins-Neto E.C., De Oliveira P.T., Beloti M.M., Rosa A.L., De Almeida A.L. (2016). Effect of surface nanotopography on bone response to titanium implant. J. Oral Implant..

[44] King S., Klineberg I., Levinger I., Brennan-Speranza T.C. (2016). The effect of hyperglycaemia on osseointegration: A review of animal models of diabetes mellitus and titanium implant placement. Arch. Osteoporos..

[45] Ramenzoni L.L., Bösch A., Proksch S., Attin T., Dds P.R.S. (2020). Effect of high glucose levels and lipopolysaccharides-induced inflammation on osteoblast mineralization over sandblasted/acid-etched titanium surface. Clin. Implant. Dent. Relat. Res..

[46] Yamawaki I., Taguchi Y., Komasa S., Tanaka A., Umeda M. (2017). Effects of glucose concentration on osteogenic differentiation of type II diabetes mellitus rat bone marrow-derived mesenchymal stromal cells on a nano-scale modified titanium. J. Periodontal Res..

[47] Burgos P.M., Rasmusson L., Meirelles L., Sennerby L. (2008). Early bone tissue responses to turned and oxidized implants in the rabbit tibia. Clin. Implant. Dent. Relat. Res..

[48] Jiang H., Ma X., Zhou W., Dong K., Rausch-Fan X., Liu S., Li S. (2017). The effects of hierarchical micro/nano-structured titanium surface on osteoblast proliferation and differentiation under diabetic conditions. Implant. Dent..

[49] Coelho P.G., Bonfante E.A., Pessoa R.S., Marin C., Granato R., Giro G., Witek L., Suzuki M. (2011). Characterization of five different implant surfaces and their effect on osseointegration: A study in dogs. J. Periodontol..

[50] Coelho P.G., Jimbo R., Tovar N., Bonfante E.A. (2015). Osseointegration: Hierarchical designing encompassing the macrometer, micrometer, and nanometer length scales. Dent. Mater..

[51] Hayashi M., Jimbo R., Lindh L., Sotres J., Sawase T., Mustafa K., Andersson M., Wennerberg A. (2012). In vitro characterization and osteoblast responses to nanostructured photocatalytic TiO_2_ coated surfaces. Acta Biomater..

[52] Tomsia A.P., Lee J.S., Wegst U.G., Saiz E. (2013). Nanotechnology for dental implants. Int. J. Oral Maxillofac. Implant..

[53] Webster T.J., Ahn E.S. (2007). Nanostructured biomaterials for tissue engineering bone. Process. Integr. Biochem. Eng..

[54] Rupp F., Liang L., Geis-Gerstorfer J., Scheideler L., Huettig F. (2018). Surface characteristics of dental implants: A review. Dent. Mater..

[55] Alexandergehrke S., Martínez C.P.-A., Piattelli A., Shibli J.A., Markovic A., Guirado J.L.C. (2016). The influence of three different apical implant designs at stability and osseointegration process: Experimental study in rabbits. Clin. Oral Implant. Res..

[56] Ajami E., Bell S., Liddell R.S., Davies J.E. (2016). Early bone anchorage to micro-and nano-topographically complex implant surfaces in hyperglycemia. Acta Biomater..

[57] Coelho P.G., Teixeira H.S., Marin C., Witek L., Tovar N., Janal M.N., Jimbo R. (2013). The in vivo effect of P-15 coating on early osseointegration. J. Biomed. Mater. Res. Part. B Appl. Biomater..

[58] Coelho P.G., Freire J.N., Granato R., Marin C., Bonfante E.A., Gil J.N., Chuang S.-K., Suzuki M. (2011). Bone mineral apposition rates at early implantation times around differently prepared titanium surfaces: A study in beagle dogs. Int. J. Oral Maxillofac. Implant..

[59] Van De Weghe S., Coelho P.G., Vanhove C., Wennerberg A., Jimbo R. (2013). Utilizing micro-computed tomography to evaluate bone structure surrounding dental implants: A comparison with histomorphometry. J. Biomed. Mater. Res. Part. B Appl. Biomater..

[60] Beck G.R., Sullivan E.C., Moran E., Zerler B. (1998). Relationship between alkaline phosphatase levels, osteopontin expression, and mineralization in differentiating MC3T3-E1 osteoblasts. J. Cell. Biochem..

[61] Stein G.S., Lian J.B., Stein J.L., Van Wijnen A.J., Montecino M. (1996). Transcriptional control of osteoblast growth and differentiation. Physiol. Rev..

[62] De Oliveira P., De Melo Soares M.S., Silveira E.S.A.M.M., Taba M., Palioto D.B., Messora M.R., Ghiraldini B., Nunes F.A.S., De Souza S.L.S. (2020). Influence of nano-hydroxyapatite coating implants on gene expression of osteogenic markers and micro-CT parameters. An in vivo study in diabetic rats. J. Biomed. Mater. Res. A.

[63] Scarano A., Lorusso F., Orsini T., Morra M., Iviglia G., Valbonetti L. (2019). Biomimetic surfaces coated with covalently immobilized collagen type i: An x-ray photoelectron spectroscopy, atomic force microscopy, micro-CT and histomorphometrical study in rabbits. Int. J. Mol. Sci..

[64] Yıldırım T.T., Dündar S., Bozoğlan A., Karaman T., Kahraman O.E., Özcan E.C. (2020). The effects of metformin on the bone filling ration around of TiAl6Va4 implants in non diabetic rats. J. Oral Biol. Craniofacial Res..

[65] Guimaraes R.P., De Oliveira P.A.D., Oliveira A.M.S.D. (2011). Effects of induced diabetes and the administration of aminoguanidine in the biomechanical retention of implants: A study in rats. J. Periodontal Res..

